# Association between mammographic breast density and histologic features of benign breast disease

**DOI:** 10.1186/s13058-017-0922-6

**Published:** 2017-12-19

**Authors:** Karthik Ghosh, Robert A. Vierkant, Ryan D. Frank, Stacey Winham, Daniel W. Visscher, Vernon S. Pankratz, Christopher G. Scott, Kathleen Brandt, Mark E. Sherman, Derek C. Radisky, Marlene H. Frost, Lynn C. Hartmann, Amy C. Degnim, Celine M. Vachon

**Affiliations:** 1 General Internal Medicine, Breast Diagnostic Clinic, Mayo Clinic, 200 First Street SW, Rochester, MN USA; 2 Health Sciences Research, Mayo Clinic, 200 First Street SW, Rochester, MN USA; 3 Anatomic Pathology, Mayo Clinic, 200 First Street SW, Rochester, MN USA; 4 Department of Internal Medicine, University of New Mexico, Albuquerque, NM USA; 5 Department of Radiology, Mayo Clinic, 200 First Street SW, Rochester, MN USA; 6 Health Sciences Research, Mayo Clinic, 4500 San Pablo Road, Jacksonville, FL USA; 7 Cancer Biology, Mayo Clinic, 4500 San Pablo Road, Jacksonville, FL 32224 USA; 8 Women’s Cancer Program, Mayo Clinic, 200 First Street SW, Rochester, MN USA; 9 Medical Oncology, Mayo Clinic, 200 First Street SW, Rochester, MN USA; 10 Breast, Endocrine, Metabolic, and GI Surgery, Mayo Clinic, 200 First Street SW, Rochester, MN USA; 11 Department of Health Sciences Research, Mayo Clinic, Minnesota BioBusiness Center 5-81, 200 First Street S.W., Rochester, MN 55904 USA

## Abstract

**Background:**

Over 40% of women undergoing breast screening have mammographically dense breasts. Elevated mammographic breast density (MBD) is an established breast cancer risk factor and is known to mask tumors within the dense tissue. However, the association of MBD with high risk benign breast disease (BBD) is unknown.

**Method:**

We analyzed data for 3400 women diagnosed with pathologically confirmed BBD in the Mayo Clinic BBD cohort from 1985–2001, with a clinical MBD measure (either parenchymal pattern (PP) or Breast Imaging Reporting and Data Systems (BI-RADS) density) and expert pathology review. Risk factor information was collected from medical records and questionnaires. MBD was dichotomized as dense (PP classification P2 or DY, or BI-RADS classification c or d) or non-dense (PP classification N1 or P1, or BI-RADS classification a or b). Associations of clinical and histologic characteristics with MBD were examined using logistic regression analysis to estimate odds ratios (ORs) with 95% confidence intervals (CIs).

**Results:**

Of 3400 women in the study, 2163 (64%) had dense breasts. Adjusting for age and body mass index (BMI), there were positive associations of dense breasts with use of hormone therapy (HT), lack of lobular involution, presence of atypical lobular hyperplasia (ALH), histologic fibrosis, columnar cell hyperplasia/flat epithelia atypia (CCH/FEA), sclerosing adenosis (SA), cyst, usual ductal hyperplasia, and calcifications. In fully adjusted multivariate models, HT (1.3, 95% CI 1.1–1.5), ALH (1.5, 95% CI 1.0–2.2), lack of lobular involution (OR 1.6, 95% CI 1.2–2.1, compared to complete involution), fibrosis (OR 2.2, 95% CI 1.9–2.6) and CCH/FEA (OR 1.3, 95% CI 1.0–1.6) remained significantly associated with high MBD.

**Conclusion:**

Our findings support an association between high risk BBD and high MBD, suggesting that risks associated with the latter may act early in breast carcinogenesis.

**Electronic supplementary material:**

The online version of this article (doi:10.1186/s13058-017-0922-6) contains supplementary material, which is available to authorized users.

## Background

Mammographic breast density (MBD) reflects the proportion of the breast composed of fibroglandular tissue and is an established risk factor for breast cancer (BC) [1, 2]. Women with the highest level of MBD are at 4–6 times increased risk compared to women with non-dense breasts. [1] Dense breasts are also associated with reduced sensitivity for detection of cancer due to masking of tumors within dense breast tissue [1].

Previous reports provide insight into the association between MBD and breast cancer, by studying the histology underlying dense tissue [3–11]. These studies included tissues diagnosed as benign breast disease (BBD) within the clinical setting and in healthy research biopsies from breasts without an underlying breast lesion. In a study of mammographically dense and non-dense core biopsies from healthy women, we reported that dense areas reflect a greater proportion of epithelium and stroma and a lesser proportion of fat when compared to non-dense tissue [12]. We and others have shown that MBD is inversely associated with lobular involution (physiologic atrophy of breast) among women with benign breast disease (BBD) [3, 4, 13]. Women with no involution or partial involution were more likely to have dense parenchymal pattern or higher percent density compared to women with complete involution. However, although correlated, not all women with complete involution had fatty or non-dense breasts; in fact, 52% of those with complete involution had dense breasts. Conversely, 23% of women with no involution had fatty or non-dense breasts. Thus, the interplay between involution and dense breasts, and associated risk, is likely complex. Since a large proportion of the screening mammography population have dense breasts [10], it is important to understand whether benign lesions identified in dense breasts are different than those in fatty breasts. We and others have reported on breast cancer risk and histologic features of BBD, finding increased cancer risk with atypical hyperplasia, sclerosing adenosis (SA), flat epithelial atypia (FEA), radial scar, and papilloma [13–18]. Understanding the types of benign breast findings in dense breast tissue would suggest that MBD could act early in carcinogenesis and also could guide aspects of screening and diagnosis for this population.

Here, we report a comprehensive evaluation of the histologic features of benign breast disease in dense and non-dense breasts. In addition, we also investigate the differential association of fibrosis and fat with lobular involution in dense breasts.

## Method

### Study cohort

The Mayo BBD cohort has been described in detail previously and includes 13,441 women, 18 to 85 years of age at biopsy, diagnosed with BBD at Mayo Clinic in Rochester, Minnesota, between 1967 and 2001 and with no prior history of breast cancer [19]. Of these, 7979 were diagnosed during the mammographic era of 1985 or later. Women with radiologic or palpable breast abnormalities who had a biopsy showing benign findings were eligible for this cohort. Risk factor information such as age, body mass index (BMI), and postmenopausal hormone therapy (HT) ever or never use, were collected from Mayo Clinic medical records, an annual clinical mammography survey, and questionnaire data obtained from study participants or their next of kin [19]. BMI information corresponded to a time closest to the breast biopsy and mammogram. Incident breast cancers were ascertained from follow-up questionnaires, clinic tumor registry, and medical record review. Only cohort participants who had a mammogram at Mayo Clinic within 6 months prior to biopsy were eligible for this breast density study (N = 4389). We further restricted the study cohort to patients with complete histological and clinical characteristics (N = 3400).

Of note, all the women in the Mayo BBD cohort had breast biopsies that showed benign findings that were concordant with imaging. If imaging and pathology results were discordant, an excisional biopsy was performed and in the event of malignancy, the patients were excluded. Hence, there were no prevalent cancers in this study.

All of the study procedures and contact materials were approved by the Mayo Clinic Institutional Review Board. Patients consenting to research authorization allow medical records, images, and cancer data to be used for research (93% response rate). Only patients who provided this consent were included in the current study.

### Mammographic breast density measures

**Fig. 1:**
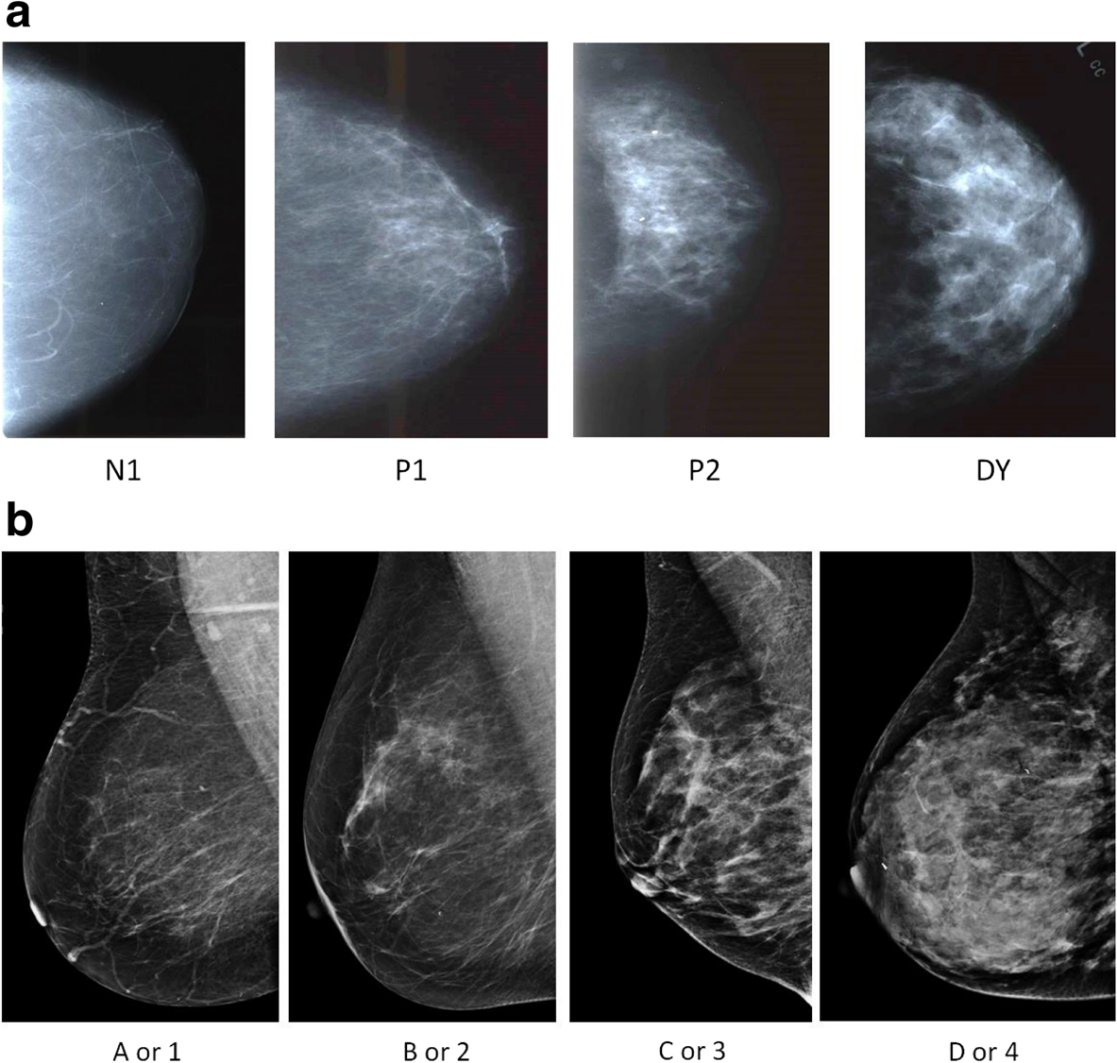
Mammographic breast density measures: **a** Parenchymal patterns. **b** Breast Imaging Reporting and Data Systems (BI-RADS) density

MBD was available from the Mayo Clinic medical record starting in 1985. From 1985 to 1996, MBD was assessed clinically by radiologists using the parenchymal pattern (PP) consisting of four categories, and based on extent and type of density (Fig. 1a): N1 (non-dense, no ducts visible), P1 (ductal prominence occupying < 25% breast), P2 (prominent ductal pattern occupying > 25% of the breast, and DY (homogenous plaque-like areas of density) [20, 21]. The parenchymal pattern measure of MBD has consistently been reported as a risk factor in studies of MBD and breast cancer risk, including our own [2] and has been shown to have modest inter-reader agreement [20, 22].

From 1997 to 2001 MBD was assessed clinically using the American College of Radiology Breast Imaging Reporting and Data System (BI-RADS) 4th edition classification (Fig. 1b): 1 (almost entirely fatty), 2 (scattered fibroglandular tissue), 3 (heterogeneously dense), and 4 (extremely dense). In the current edition, these are now categorized from “a” to “d” [23]. The BI-RADS density measure has also been consistently associated with breast cancer [24].

The lack of one-to-one correspondence between PP and BI-RADS definitions did not allow us to retain the 4-level classifications when combining the measures. However, it is generally accepted that the bottom two categories of each correspond to lower density and the top two categories to higher density. Thus, for the purposes of this 16-year study during which the density measure changed from PP to BI-RADS, the clinical density measures were categorized into dense (PP P2 or DY or BI-RADS c or d) or non-dense (PP N1 or P1 or BI-RADS a or b).

### Histology of breast biopsy

**Fig. 2:**
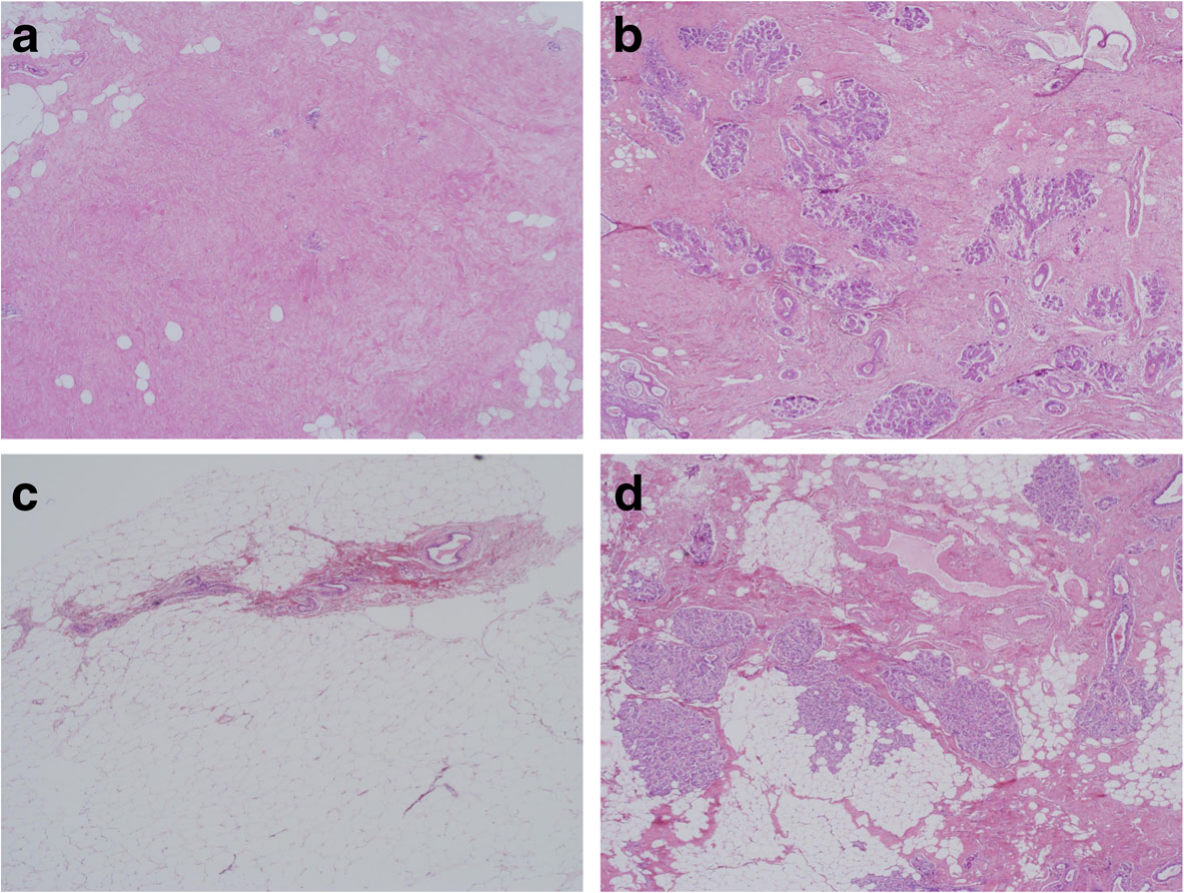
Histology of breast tissue showing combinations of fibrosis (“no” for absent and “yes” for present) and extent of lobular involution (no involution and complete involution). **a** Fibrosis “yes”, involution complete. **b** Fibrosis “yes”, involution “no”. **c** Fibrosis “no”, involution complete. **d** Fibrosis “no”, involution “no”

All histologic assessments of benign breast tissue were performed by one expert breast pathologist masked to the patient’s MBD and BBD reports. The pathologist (DV) assessed the overall histologic impression of the benign breast lesion as non-proliferative disease (NP), proliferative disease without atypia (PDWA), and atypical hyperplasia (atypical ductal hyperplasia or ADH, and atypical lobular hyperplasia or ALH). In addition, specific characteristics of the tissue, including columnar cell hyperplasia (CCH), flat epithelial atypia (FEA), sclerosing adenosis (SA), cyst, usual ductal hyperplasia (UDH), calcification, fibroadenoma, intra-ductal papilloma, radial scar, duct ectasia, and mucocele-like lesions, were recorded as previously described [14–17, 25, 26]. We combined CCH and FEA as they both represented proliferative columnar cell changes in breast tissue. The pathologist also classified the proportion of normal lobules on the slide showing age-related involution, as no involution (0% involution), partial (1–74% involuted lobules), or complete (≥75% involuted lobules) [18]. Fibrosis was defined as presence of dense collagen dispersed in interlobular tissues and encompassing epithelial parenchyma in an area that measured at least one × 4 microscopic field. Figure 2a–d shows histologic examples of tissue with or without involution and fibrosis.

### Statistical analysis

Data were summarized using frequencies and percentages for categorical variables, and means and standard deviations for continuous variables. The association of the frequency of missing data with patient characteristics was assessed using the chi-square test for categorical variables and Wilcoxon rank sum test for continuous variables. Multivariate stepwise logistic regression analysis was then used to determine independent predictors of missingness after accounting for the effects of other variables.

We examined associations between having a dense breast and demographic, clinical, and histologic characteristics using logistic regression analysis. The following characteristics were examined: age at biopsy, body mass index (BMI) at biopsy, ever use of HT, extent of lobular involution, and presence vs. absence of ADH or ALH, fibrosis, SA, CCH/FEA, cysts, UDH, calcifications, fibroadenoma, intra-ductal papilloma, radial scars, duct ectasia, and mucocele-like lesions. Because of our a priori interest, we also examined associations beween dense breasts and combinations of the extent of lobular involution and fibrosis (stromal replacement). For each variable described above, we first fit a series of age-adjusted and BMI-adjusted models, examining associations between each of the characteristics of interest and dense vs. non-dense MBD. All such variables found to be statistically significant (*p* < 0.05), were then included in a final, multivariate model. Histologic impression was not included in this final model because it is defined in large part based on the individual histologic characteristics that were examined. Primary analyses were of associations with MBD in the entire group of subjects. Secondary analyses included re-examination of the associations within strata defined by ever vs. never using HT, premenopausal vs. postmenopausal at biopsy, and type of density measure (BI-RADS vs. PP). All statistical tests were two-sided, and all analyses were carried out using the SAS software system (SAS Institute, Inc., Cary, NC, USA).

## Results

Compared to women in the cohort who were excluded because of missing information, women in the analysis were older, were more likely to have progressed to breast cancer following their benign biopsy, had a slightly higher BMI, were followed for a longer period of time, were more likely to have had an excisional biopsy rather than a core needle biopsy, were more likely to have had a mammographically detected lesion rather than a palpable lump, had a history of HT use and were more likely to have specific histopathologic features, including incomplete involution, fibrosis, CCH/FEA, cyst, and sclerosing adenosis in the benign lesion (Additional file 1: Table S1). Ascertainment based on year of biopsy was also strongly associated with inclusion in the cohort. In a multivariate model, the  only variables that remained significantly associated with study inclusion were year of biopsy, eventual breast cancer status, length of follow up, presence of ALH, and indication for biopsy.

For the 3400 eligible women, the median age at biopsy was 53 years, with 13% aged < 40 years, 53% between 40 and 59 years, and 34% ≥ 60 years. Median BMI was 26, and 61% were current or past HT users. In this sample, 56% of women had non-proliferative disease, 38% had PDWA, and 6% had AH. Further, 64% had dense breasts (60% using BI-RADS density of “c” or “d”; 65% with PP as P2 or DY). Associations between MBD and histologic characteristics are presented in Table 1. As shown in the table, younger age, lower BMI, ever use of HT, and lesser extent of lobular involution at biopsy were associated with having dense breasts. Presence of ALH, fibrosis, CCH/FEA, SA, cysts, moderate to florid UDH, and calcifications were associated with having a dense breast in models adjusted for age and BMI (*p* < 0.01). With multivariate adjustments, age, BMI, HT, ALH, lobular involution, fibrosis, and CCH/FEA were statistically significantly associated with dense breasts (*p* ≤ 0.05). Presence of fibrosis was associated with a 2-fold increased risk of having dense breasts (OR = 2.2, 95% CI 1.9–2.6) and no involution with a 1.6-fold increased risk relative to complete involution (OR = 1.6, 95% CI 1.2–2.1). CCH/FEA was positively associated with dense breasts (OR = 1.3, 95% CI 1.0–1.7; *p* = 0.036) (Table 1). Women with a diagnosis of ALH had a 50% greater likelihood of having dense breasts than those without (OR 1.5, 95% CI 1.2–2.1, *p* = 0.05).

**Table 1:** Association between mammographic breast density and benign breast findings

				Age-adjusted and BMI-adjusted		Fully adjusted^a^	
Covariate	Total N = 3400	Non-dense N = 1237	Dense N = 2163	Odds ratio (95% CI)	*p* value^b^	Odds ratio (95% CI)	*P* value
Age of BBD					<.001		<.001
Ordinal effect	3400	57.7 (12.7)	51.7 (12.5)	0.96 (0.96, 0.97)		0.96 (0.96, 0.97)	
BMI at biopsy					<.001		<.001
Ordinal effect	3400	28.8 (7.0)	25.8 (5.3)	0.92 (0.91, 0.93)		0.93 (0.91, 0.94)	
HT ever/never					<.001		0.002
No	1341	529 (39.4%)	812 (60.6%)	1.00 (ref)		1.00 (ref)	
Yes	2059	708 (34.4%)	1351 (65.6%)	1.30 (1.12, 1.52)		1.29 (1.10, 1.50)	
ADH					0.896		
Absent	3339	1213 (36.3%)	2126 (63.7%)	1.00 (ref)			
Present	61	24 (39.3%)	37 (60.7%)	1.04 (0.60, 1.78)			
ALH					0.001		0.050
Absent	3246	1196 (36.8%)	2050 (63.2%)	1.00 (ref)		1.00 (ref)	
Present	154	41 (26.6%)	113 (73.4%)	1.88 (1.28, 2.74)		1.49 (1.00, 2.21)	
Involution					<.001		0.006
Complete	992	479 (48.3%)	513 (51.7%)	1.00 (ref)		1.00 (ref)	
Partial	1920	644 (33.5%)	1276 (66.5%)	1.36 (1.14, 1.61)		1.03 (0.86, 1.25)	
None	488	114 (23.4%)	374 (76.6%)	1.57 (1.18, 2.07)		1.55 (1.16, 2.07)	
Fibrosis					<.001		<.001
Absent	1233	605 (49.1%)	628 (50.9%)	1.00 (ref)		1.00 (ref)	
Present	2167	632 (29.2%)	1535 (70.8%)	2.27 (1.95, 2.65)		2.22 (1.87, 2.62)	
CCH/FEA					<.001		0.036
Absent	2273	894 (39.3%)	1379 (60.7%)	1.00 (ref)		1.00 (ref)	
Present	1127	343 (30.4%)	784 (69.6%)	1.71 (1.45, 2.01)		1.30 (1.02, 1.66)	
Sclerosing adenosis					<.001		0.531
Absent	2269	899 (39.6%)	1370 (60.4%)	1.00 (ref)		1.00 (ref)	
Present	1131	338 (29.9%)	793 (70.1%)	1.59 (1.35, 1.86)		1.07 (0.86, 1.33)	
Cyst					<.001		0.981
Absent	1379	557 (40.4%)	822 (59.6%)	1.00 (ref)		1.00 (ref)	
Present	2021	680 (33.6%)	1341 (66.4%)	1.45 (1.24, 1.68)		1.00 (0.84, 1.19)	
Usual ductal hyperplasia					<.001		0.544
None	1984	750 (37.8%)	1234 (62.2%)	1.00 (ref)		1.00 (ref)	
Mild	493	179 (36.3%)	314 (63.7%)	1.15 (0.93, 1.43)		0.87 (0.69, 1.09)	
Moderate	678	227 (33.5%)	451 (66.5%)	1.54 (1.26, 1.87)		1.05 (0.83, 1.34)	
Florid	245	81 (33.1%)	164 (66.9%)	1.62 (1.20, 2.19)		0.98 (0.69, 1.38)	
Calcifications					<.001		0.325
Absent	1738	647 (37.2%)	1091 (62.8%)	1.00 (ref)		1.00 (ref)	
Present	1662	590 (35.5%)	1072 (64.5%)	1.34 (1.15, 1.56)		1.09 (0.92, 1.29)	
Fibroadenoma					0.065		
Absent	2433	864 (35.5%)	1569 (64.5%)	1.00 (ref)			
Present	966	372 (38.5%)	594 (61.5%)	0.86 (0.73, 1.01)			
Intra-ductal papilloma					0.111		
Absent	3128	1138 (36.4%)	1990 (63.6%)	1.00 (ref)			
Present	262	94 (35.9%)	168 (64.1%)	1.25 (0.95, 1.66)			
Radial scars					0.052		
Absent	3168	1166 (36.8%)	2002 (63.2%)	1.00 (ref)			
Present	230	71 (30.9%)	159 (69.1%)	1.35 (1.00, 1.82)			
Duct ectasia					0.440		
Absent	2917	1065 (36.5%)	1852 (63.5%)	1.00 (ref)			
Present	481	172 (35.8%)	309 (64.2%)	1.09 (0.88, 1.34)			
Mucocele-like lesions					0.559		
Absent	3356	1224 (36.5%)	2132 (63.5%)	1.00 (ref)			
Present	39	13 (33.3%)	26 (66.7%)	1.23 (0.61, 2.50)			

*BMI* body mass index, *BBD* benign breast disease, *HT* hormone therapy, *ADH* atypical ductal hyperplasia, *ALH* atypical lobular hyperplasia, *CCH/FEA* columnar cell hyperplasia/flat epithelial atypia

^a^Adjusted for all covariates ^b^Remained significant in age-adjusted and BMI-adjusted model

**Fig. 3:**
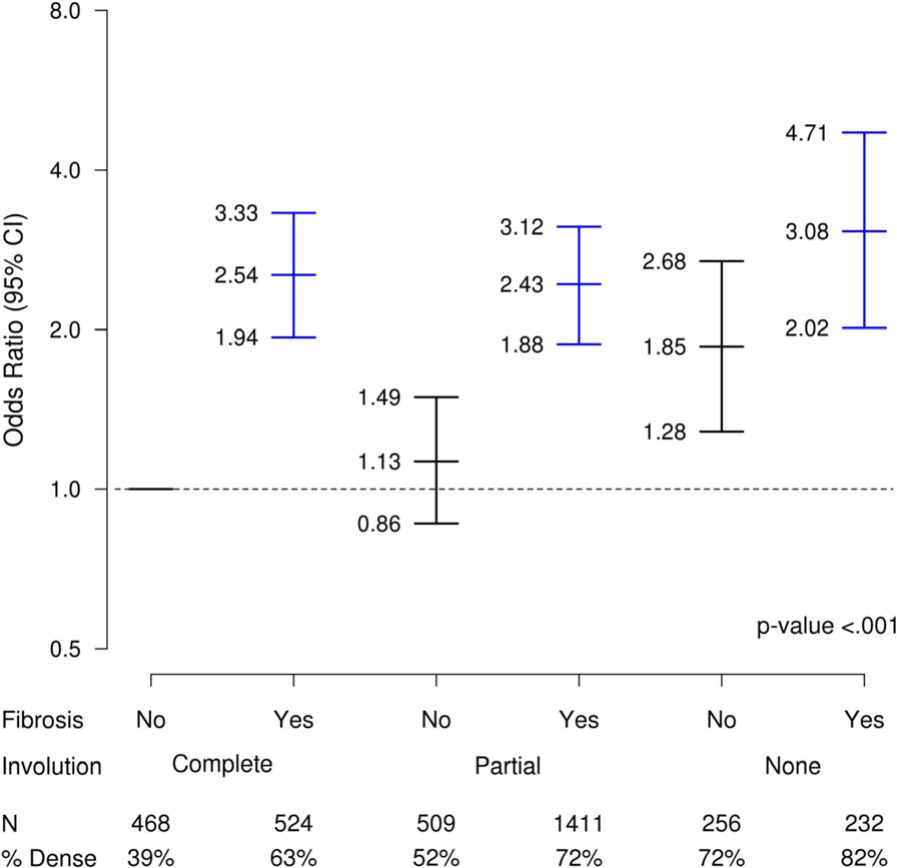
Association between dense breasts and combinations of fibrosis (“no” for absent and “yes” for present) and extent of lobular involution (complete, partial, or none). Referent group is women with no fibrosis and complete involution. Results are adjusted for age at initial biopsy and body mass index at initial biopsy

Associations between MBD and combinations of lobular involution and fibrosis are presented in Table 2 and Fig. 3. Age-adjusted and BMI-adjusted analyses revealed that women with incomplete involution and/or fibrosis were significantly more likely to have high MBD than those without fibrosis and with complete involution (*p* < 0.05 for all combinations). In particular, after adjustment for age and BMI, women with no involution and with fibrosis were more than three times as likely to have dense breasts as those with complete involution and no fibrosis (OR 3.5, 95% CI 2.3–5.3). These associations were attenuated somewhat, but still remained highly significant after multivariate adjustment. There was no statistically significant interaction between involution and fibrosis (*p* > 0.2 in models adjusted for age and BMI and multivariate models), indicating that the marginal effects of each variable on the odds of having dense breasts are not modified by the other.

**Table 2:** Association of density (combined BI-RADS and Parenchymal Pattern) with fibrosis and involution combination

			Age-adjusted and BMI-adjusted		Fully adjusted^a^	
Covariate	Total N = 3400	Events N = 2163	Odds ratio (95% CI)	*p* value	Odds ratio (95% CI)	*p* value
Involution/Fibrosis				<.001		<.001
Complete/No	468	183 (39.1%)	1.00 (ref)		1.00 (ref)	
Complete/Yes	524	330 (63.0%)	2.46 (1.88, 3.20)		2.54 (1.94, 3.33)	
Partial/No	509	262 (51.5%)	1.19 (0.91, 1.56)		1.13 (0.86, 1.49)	
Partial/Yes	1411	1014 (71.9%)	2.73 (2.17, 3.44)		2.43 (1.88, 3.12)	
None/No	256	183 (71.5%)	1.83 (1.27, 2.63)		1.85 (1.28, 2.68)	
None/Yes	232	191 (82.3%)	3.52 (2.33, 5.30)		3.08 (2.02, 4.71)	

*BI-RADS* Breast Imaging Reporting and Data Systems, *BMI* body mass index

^a^Includes all covariates from Table 1 that remained statistically significant after age and BMI adjustment

Results were similar when examining associations within subsets of women who had ever vs. never taken HT and premenopausal vs. postmenopausal status (data not shown). Results were also similar when examining the associations in a subset of women with BI-RADS (Additional file 2: Table S2) and PP measures (Additional file 3: Table S3), with the only possible exception being that presence of cysts was associated with non-dense breasts in multivariate analyses of BI-RADS measures (OR = 0.68, 95% CI 0.47–0.97, *p* = 0.036), but was null for PP measures (OR = 1.17, 95% CI 0.81–1.68, *p* = 0.138). However, the power was limited for these comparisons.

## Discussion

We performed a comprehensive investigation of the association between mammographic breast density and specific histologic findings in benign breast tissue. Among women with benign breast disease, having dense breasts was associated with fibrosis and lack of lobular involution. Further, high-risk lesions such as ALH and CCH/FEA were found to be increased in biopsies from women with dense breasts compared to women with non-dense breasts.

In a subset of the current population with parenchymal pattern measures, we previously showed that age-related lobular involution was inversely associated with MBD [13] and that MBD and lobular involution were independently associated with breast cancer risk [3]. While increasing MBD was associated with increased BC risk, increasing lobular involution was associated with decreasing BC risk [1–4, 18]. These results are consistent with those of Gierach et al. [4], who showed an association between reduced measures of terminal duct lobular unit (TDLU) involution on benign breast biopsies and higher MBD especially among premenopausal women. They reported that in premenopausal women, the TDLU count was associated with percent perilesional MBD, and reduced TDLU involution was associated with higher perilesional area and volumetric density, thereby suggesting that the association between MBD and BC risk may be related to some extent with the epithelial component of the breast. We, however, did not find differences by menopausal status. It is notable possibly that the analysis of Gierach et al. was limited to women ages 40–65 years with radiologically detected lesions, who were diagnosed between 2007 and 2010, as compared with the wider age range and broader biopsy indications among participants in the Mayo BBD cohort.

With regard to the stromal component, our current study results also showed that fibrosis is more commonly seen in dense breasts compared to non-dense breasts. These findings are concordant with several prior studies showing that dense tissue is associated with stromal tissue in the breast [5, 6, 12, 27]. Li et al. performed quantitative microscopy studies on breast tissue obtained from forensic autopsy showing that percent MBD is associated with nuclear area, both epithelial and non-epithelial, collagen, and area of glandular tissue [6]. They also showed that image-guided sampling from breast tissue with high MBD had higher stroma and lower fat compared to low MBD regions within the same breast. Huo et al. identified stromal collagen deposition and organization in tissue from high-density breasts compared to low-density breasts [7]. Keely and colleagues have shown in mouse mammary models that higher stromal collagen density was associated with greater tumor burden and more invasive types of cancer, suggesting that stromal collagen density may have a link with breast cancer initiation and progression [8]. Proposed mechanisms include increased matrix stiffness and the production of growth factors (insulin-like growth factor (IGF), epidermal growth factor (EGF) and transforming growth factor (TGF) beta) [8]. McConnell et al. studied tissue from 22 postmenopausal women and showed that stiffening of stromal collagen promotes high MBD and that the abnormal mechanical environment can initiate pathways to stimulate neoplastic changes within the epithelium [9]. Hence, stromal biology potentially influences epithelial changes that ultimately may lead to cancer. Further understanding of these changes at the molecular level can help us clarify the driving forces behind the association between MBD and breast cancer risk.

We hypothesized that fibrosis would modify the association between involution and density, such that the stromal replacement (complete involution with fibrosis) would actually have a positive association, and involution with greater proportions of fat (fat replacement) would have an inverse association. Although both fibrosis and involution were both strongly associated with density, we found no evidence of effect modification; specifically, the association between fibrosis and density was similar across all levels of involution. The greatest risk of dense breasts was among women with no involution and fibrosis in the breast tissue. Together, these findings suggest the possibility that stromal factors may influence breast epithelium, thereby predisposing it to changes leading to malignancy in women with dense breasts.

Approximately 40% of women who have had screening mammography have dense breasts classified as BI-RADS c and d [10]. In our study, 64% of the women had dense breasts, likely due to the fact that women with dense breasts have more breast biopsies [11]. We, therefore, assessed whether specific benign breast findings were more common in dense breast tissue in order to assist clinical management of women with dense breasts. In a prior report from the National Breast Screening Study, proliferative lesions with or without atypia were noted to be more common in women with dense breasts compared to those with non-dense breasts [28, 29]. We were able to examine specific types of atypical hyperplasia and did not find an association between ADH and dense breasts (*p* = 0.9) but did detect a borderline significant association between ALH and dense breasts (*p* = 0.05). Since ours is the first report to examine the type of atypical hyperplasia with dense breasts, this finding warrants further evaluation in future studies.

We also noted a positive association between MBD and CCH/FEA. In the literature, columnar cell lesions have been reported to include changes such as proliferation of columnar shaped epithelial cells within enlarged TDLUs and have been classified with or without atypia [30, 31]. Turasvili et al. conducted a study of histologic features from bilateral subcutaneous mastectomies from forensic autopsy, and reported an association between columnar cell (CC) lesions and radiographic breast density measured as high Faxitron Wolfe density (OR = 2.1, 95% CI 1.01–4.6; *p* = 0.04) [29]. They also showed that CC lesions are associated with higher tissue collagen, suggesting that stromal composition and epithelial stromal interaction may potentially contribute to the association between CC lesions and breast density. In a study of 282 women with FEA in a cohort with benign breast disease, FEA has been associated with AH in half the cases, with ADH more commonly so than ALH [25]. Further research is warranted to understand the molecular mechanisms underlying early epithelial changes in mammary glandular tissue that may provide insight into mechanisms for breast cancer development.

Our study of benign breast characteristics and breast density is the largest and most comprehensive analysis to date and based on a well-established cohort of BBD. It confirms and extends prior work in this area and is the first study to examine the interaction between involution and fibrosis. Further, this study utilized two different but validated measures of breast density and had similar findings. As the study was performed among women with BBD, the study results are generalizable to the sizable population of women who have had benign breast biopsies who have tissue available to evaluate benign characteristics. Women with BBD are considered at elevated risk compared with the general population [19]. In this report, our goal was to understand tissue changes associated with breast density in the setting of benign breast tissue. We recognize that the results are limited to women with BBD and, in general, a higher-risk population.

Our study had additional limitations. We acknowledge the occurrence of inter-observer variability in diagnosing BBD microscopically [32]. However, we have previously reported on inter-observer variability in a sample of 189 women with atypical hyperplasia from the BBD cohort and showed concordance in 87% with the initial read and after review of the discordant results again by the readers, the concordance was 97% [33]. We also noted that the women in the cohort when compared to those excluded due to lack of density information, were older, had higher BMI, but also used HT, which may have contributed to incomplete involution, fibrosis, CCH/FEA, cyst, and sclerosing adenosis being more common in this group. We also acknowledge that management of breast concerns, palpable and imaging-detected lesions has evolved over the years, from the 1980s to the current era, with core-needle biopsies becoming the standard of care for sampling breast abnormalities. Moreover, breast density measures were missing for a proportion of the cohort, more so in the later years, for women followed for a shorter period of time, and for women with palpable lesions rather than mammographically detected lesions. Continued efforts using tissue from core-needle biopsies in the more recent era may provide additional clarity to these findings. This study of benign breast tissue provides insight into the biology of breast tissue in dense and non-dense areas of the breast among women with BBD.

## Conclusion

In examining the association between benign breast changes and MBD, we report that in addition to the known association between MBD with fibrosis and lack of lobular involution, women with dense breasts may be more likely to have some high-risk lesions such as ALH and CCH/FEA compared to women with non-dense breasts. Continued research on mammary epithelial changes and of molecular markers in dense tissue is needed to shed further light on MBD so that targeted efforts on reducing breast cancer risk factors can occur.

## Additional files

Additional file 1: Table S1. Distributions of subject characteristics by study inclusion among women enrolled in the BBD study from 1985 to 2001. (DOCX 21 kb)

Additional file 2: Table S2. Association between mammographic breast density and benign breast findings for the 728 women with BI-RADS density measures. (DOCX 20 kb)

Additional file 3: Table S3. Association between mammographic breast density and benign breast findings for the 2672 women with parenchymal pattern density measures. (DOCX 20 kb)

## Abbreviations

ADH: Atypical ductal hyperplasia
ALH: Atypical lobular hyperplasia
BBD: Benign breast disease
BC: Breast cancer
BI-RADS: Breast Imaging Reporting and Data Systems
BMI: Body mass index
CCH/FEA: Columnar cell hyperplasia/flat epithelial atypia
CI: Confidence interval
EGF: Epidermal growth factor
HT: Hormone therapy
IGF1: Insulin-like growth factor 1
MBD: Mammographic breast density
NP: Non proliferative
OR: Odds ratio
PDWA: Proliferative disease without atypia
PP: Parenchymal pattern
SA: Sclerosing adenosis
TDLU: Terminal duct lobular unit
TGF beta: Transforming growth factor beta
UDH: Usual ductal hyperplasia
