# Supplementary material for: Association between mammographic breast density and histologic features of benign breast disease
Source: Breast Cancer Res. 2017 Dec 19;19:134. doi: 10.1186/s13058-017-0922-6 (PMC5735506; doi:10.1186/s13058-017-0922-6)
Supplement: Supplementary file 1 — Distributions of subject characteristics by study inclusion among women enrolled in the BBD study from 1985 to 2001. (DOCX 21 kb) [file 13058_2017_922_MOESM1_ESM.docx]

**Table S1**. Distributions of subject characteristics by study inclusion* among women enrolled in the BBD study from 1985-2001

| Covariate | Not Included N=4579 | Included * N=3400 | Total N=7979 | p-value | p-value** |
| --- | --- | --- | --- | --- | --- |
| **Age of BBD** |  |  |  | <0.001 ^†^ | - |
| Median (Q1, Q3) | 51 (41, 64) | 53 (44, 64) | 52 (42, 64) |  |  |
| **Breast Cancer Status** |  |  |  | 0.009 ^‡^ | <0.001 |
| Unaffected | 4246 (92.7%) | 3098 (91.1%) | 7344 (92.0%) |  |  |
| Affected | 333 (7.3%) | 302 (8.9%) | 635 (8.0%) |  |  |
| **Years of follow-up** |  |  |  | <0.001 ^†^ | 0.003 |
| Median (Q1, Q3) | 13.6 (9.2, 17.4) | 15.4 (12.1, 20.1) | 14.4 (10.4, 18.3) |  |  |
| **BMI at biopsy** |  |  |  | 0.059 ^†^ | - |
| Median (Q1, Q3) | 25 (23, 29) | 26 (23, 30) | 26 (23, 30) |  |  |
| **HRT ever/never** |  |  |  | <0.001 ^‡^ | - |
| Missing | 1409 | 0 | 1409 |  |  |
| No | 1580 (49.8%) | 1341 (39.4%) | 2921 (44.5%) |  |  |
| Yes | 1590 (50.2%) | 2059 (60.6%) | 3649 (55.5%) |  |  |
| **Overall impression** |  |  |  | <0.001 ^‡^ | - |
| NP | 2703 (59.0%) | 1897 (55.8%) | 4600 (57.7%) |  |  |
| PDWA | 1522 (33.2%) | 1294 (38.1%) | 2816 (35.3%) |  |  |
| AH | 354 (7.7%) | 209 (6.1%) | 563 (7.1%) |  |  |
| **ADH** |  |  |  | <0.001 ^‡^ | - |
| Absent | 4344 (94.9%) | 3339 (98.2%) | 7683 (96.3%) |  |  |
| Present | 235 (5.1%) | 61 (1.8%) | 296 (3.7%) |  |  |
| **ALH** |  |  |  | 0.003 ^‡^ | 0.031 |
| Absent | 4431 (96.8%) | 3246 (95.5%) | 7677 (96.2%) |  |  |
| Present | 148 (3.2%) | 154 (4.5%) | 302 (3.8%) |  |  |
| **Involution** |  |  |  | <0.001 ^‡^ | - |
| Complete | 1598 (34.9%) | 992 (29.2%) | 2590 (32.5%) |  |  |
| Partial | 2159 (47.2%) | 1920 (56.5%) | 4079 (51.1%) |  |  |
| None | 822 (18.0%) | 488 (14.4%) | 1310 (16.4%) |  |  |
| **Fibrosis** |  |  |  | <0.001 ^‡^ | - |
| Missing | 276 | 0 | 276 |  |  |
| Absent | 2211 (51.4%) | 1233 (36.3%) | 3444 (44.7%) |  |  |
| Present | 2092 (48.6%) | 2167 (63.7%) | 4259 (55.3%) |  |  |
| **CCH / FEA** |  |  |  | <0.001 ^‡^ | - |
| Absent | 3327 (72.7%) | 2273 (66.9%) | 5600 (70.2%) |  |  |
| Present | 1252 (27.3%) | 1127 (33.1%) | 2379 (29.8%) |  |  |
| **Sclerosing Adenosis** |  |  |  | <0.001 ^‡^ | - |
| Missing | 10 | 0 | 10 |  |  |
| absent | 3417 (74.8%) | 2269 (66.7%) | 5686 (71.4%) |  |  |
| present | 1152 (25.2%) | 1131 (33.3%) | 2283 (28.6%) |  |  |
| **Cyst** |  |  |  | <0.001 ^‡^ | - |
| Missing | 6 | 0 | 6 |  |  |
| Absent | 2593 (56.7%) | 1379 (40.6%) | 3972 (49.8%) |  |  |
| Present | 1980 (43.3%) | 2021 (59.4%) | 4001 (50.2%) |  |  |
| **Usual ductal hyperplasia** |  |  |  | <0.001 ^‡^ | - |
| Missing | 248 | 0 | 248 |  |  |
| None | 3011 (69.5%) | 1984 (58.4%) | 4995 (64.6%) |  |  |
| Mild | 464 (10.7%) | 493 (14.5%) | 957 (12.4%) |  |  |
| Moderate | 624 (14.4%) | 678 (19.9%) | 1302 (16.8%) |  |  |
| Florid | 232 (5.4%) | 245 (7.2%) | 477 (6.2%) |  |  |
| **Calcifications** |  |  |  | <0.001 ^‡^ | - |
| Missing | 12 | 0 | 12 |  |  |
| Absent | 2795 (61.2%) | 1738 (51.1%) | 4533 (56.9%) |  |  |
| Present | 1772 (38.8%) | 1662 (48.9%) | 3434 (43.1%) |  |  |
| **Fibroadenoma** |  |  |  | <0.001 ^‡^ | - |
| Missing | 2 | 1 | 3 |  |  |
| Absent | 3045 (66.5%) | 2433 (71.6%) | 5478 (68.7%) |  |  |
| Present | 1532 (33.5%) | 966 (28.4%) | 2498 (31.3%) |  |  |
| **Intra-ductal papilloma** |  |  |  | <0.001 ^‡^ | - |
| Missing | 11 | 10 | 21 |  |  |
| Absent | 4097 (89.7%) | 3128 (92.3%) | 7225 (90.8%) |  |  |
| Present | 471 (10.3%) | 262 (7.7%) | 733 (9.2%) |  |  |
| **Radial scars** |  |  |  | 0.007 ^‡^ | - |
| Missing | 3 | 2 | 5 |  |  |
| Absent | 4332 (94.7%) | 3168 (93.2%) | 7500 (94.1%) |  |  |
| Present | 244 (5.3%) | 230 (6.8%) | 474 (5.9%) |  |  |
| **Duct Ectasia** |  |  |  | 0.002 ^‡^ | - |
| Missing | 125 | 2 | 127 |  |  |
| Absent | 3928 (88.2%) | 2917 (85.8%) | 6845 (87.2%) |  |  |
| Present | 526 (11.8%) | 481 (14.2%) | 1007 (12.8%) |  |  |
| **Mucocele like tumors** |  |  |  | 0.224 ^‡^ | - |
| Missing | 10 | 5 | 15 |  |  |
| Absent | 4529 (99.1%) | 3356 (98.9%) | 7885 (99.0%) |  |  |
| Present | 40 (0.9%) | 39 (1.1%) | 79 (1.0%) |  |  |
| **Year of biopsy** |  |  |  | <0.001 ^‡^ | <0.001 |
| 1985-1989 | 1194 (26.1%) | 1521 (44.7%) |  |  |  |
| 1990-1994 | 1162 (25.4%) | 1042 (30.7%) |  |  |  |
| 1995-1999 | 1702 (37.2%) | 528 (15.5%) |  |  |  |
| 2000-2001 | 521 (11.4%) | 309 (9.1%) |  |  |  |
| **Biopsy type** |  |  |  | <0.001 ^‡^ | - |
| Excisional | 3246 (70.9%) | 2830 (83.2%) | 6076 (76.1%) |  |  |
| Core | 1333 (29.1%) | 570 (16.8%) | 1903 (23.9%) |  |  |
| **Indication for biopsy** |  |  |  | <0.001 ^‡^ | <0.001 |
| Missing | 1175 | 280 | 1455 |  |  |
| Lump | 1528 (44.9%) | 1087 (34.8%) | 2615 (40.1%) |  |  |
| Mammogram | 1876 (55.1%) | 2033 (65.2%) | 3909 (59.9%) |  |  |
| * A woman was included in the study if she had a mammogram at Mayo Clinic within six months of initial biopsy and had complete histological and clinical information available on covariates significantly associated with density after age and bmi adjustment. ^†^ Wilcoxon rank sum test    ^‡^ Chi-square test ** Stepwise logistic regression analysis | | | | |  |
