# Supplementary material for: Association between mammographic breast density and histologic features of benign breast disease
Source: Breast Cancer Res. 2017 Dec 19;19:134. doi: 10.1186/s13058-017-0922-6 (PMC5735506; doi:10.1186/s13058-017-0922-6)
Supplement: Supplementary file 2 — Association between mammographic breast density and benign breast findings for the 728 women with BI-RADS density measures. (DOCX 20 kb) [file 13058_2017_922_MOESM2_ESM.docx]

**Table S2.** Association between mammographic breast density and benign breast findings for the 728 women with BI-RADS density measures.

|  | |  |  |  | **Age- and BMI-Adjusted** | | **Fully Adjusted*** | |  |  |  |
| --- | --- | --- | --- | --- | --- | --- | --- | --- | --- | --- | --- |
| Covariate | | Non-Dense N=294 | Dense N=434 | Total N=728 | Odds Ratio  (95% CI) | p-value | Odds Ratio  (95% CI) | p-value |  |  |  |
| **Age of BBD** | |  |  |  |  | <0.001 |  | <0.001 |  |  |  |
| Mean (SD) | | 58.3 (12.0) | 53.5 (11.9) | 55.4 (12.1) | 0.96 (0.95-0.98) |  | 0.97 (0.95-0.98) |  |  |  |  |
| **BMI at biopsy** | |  |  |  |  | <0.001 |  | <0.001 |  |  |  |
| Mean (SD) | | 30.6 (9.5) | 26.8 (6.2) | 28.3 (7.9) | 0.92 (0.90-0.94) |  | 0.93 (0.91-0.96) |  |  |  |  |
| **HRT ever/never** | |  |  |  |  | 0.038 |  | 0.063 |  |  |  |
| No | | 113 (38.4%) | 155 (35.7%) | 268 (36.8%) | 1.00  (ref) |  | 1.00  (ref) |  |  |  |  |
| Yes | | 181 (61.6%) | 279 (64.3%) | 460 (63.2%) | 1.44 (1.02-2.03) |  | 1.41 (0.98-2.01) |  |  |  |  |
| **ADH** | |  |  |  |  | 0.959 |  |  |  |  |  |
| Absent | | 293 (99.7%) | 432 (99.5%) | 725 (99.6%) | 1.00  (ref) |  |  |  |  |  |  |
| Present | | 1 (0.3%) | 2 (0.5%) | 3 (0.4%) | 1.07 (0.08-14.96) |  |  |  |  |  |  |
| **ALH** | |  |  |  |  | <0.001 |  | 0.305 |  |  |  |
| Absent | | 283 (96.3%) | 402 (92.6%) | 685 (94.1%) | 1.00  (ref) |  | 1.00  (ref) |  |  |  |  |
| Present | | 11 (3.7%) | 32 (7.4%) | 43 (5.9%) | 1.99 (0.96-4.15) |  | 1.50 (0.69-3.24) |  |  |  |  |
| **Involution** | |  |  |  |  | 0.008 |  | 0.051 |  |  |  |
| Complete | | 162 (55.1%) | 154 (35.5%) | 316 (43.4%) | 1.00  (ref) |  | 1.00  (ref) |  |  |  |  |
| Partial | | 107 (36.4%) | 199 (45.9%) | 306 (42.0%) | 0.92 (0.90-0.95) |  | 1.44 (0.99-2.09) |  |  |  |  |
| None | | 25 (8.5%) | 81 (18.7%) | 106 (14.6%) | 1.59 (1.12-2.25) |  | 1.89 (1.06-3.36) |  |  |  |  |
| **Fibrosis** | |  |  |  |  | <0.001 |  | <0.001 |  |  |  |
| Absent | | 221 (75.2%) | 243 (56.0%) | 464 (63.7%) | 1.00  (ref) |  | 1.00  (ref) |  |  |  |  |
| Present | | 73 (24.8%) | 191 (44.0%) | 264 (36.3%) | 2.19 (1.55-3.09) |  | 2.23 (1.56-3.19) |  |  |  |  |
| **CCH / FEA** | |  |  |  |  | 0.002 |  | 0.040 |  |  |  |
| Absent | | 211 (71.8%) | 263 (60.6%) | 474 (65.1%) | 1.00  (ref) |  | 1.00  (ref) |  |  |  |  |
| Present | | 83 (28.2%) | 171 (39.4%) | 254 (34.9%) | 1.69 (1.21-2.37) |  | 1.65 (1.02-2.67) |  |  |  |  |
| **Sclerosing Adenosis** | |  |  |  |  | 0.067 |  | 0.759 |  |  |  |
| absent | | 238 (81.0%) | 321 (74.0%) | 559 (76.8%) | 1.00  (ref) |  | 1.00  (ref) |  |  |  |  |
| present | | 56 (19.0%) | 113 (26.0%) | 169 (23.2%) | 1.43 (0.98-2.08) |  | 0.93 (0.58-1.49) |  |  |  |  |
| **Cyst** | |  |  |  |  | 0.959 |  | 0.036 |  |  |  |
| Absent | | 162 (55.1%) | 233 (53.7%) | 395 (54.3%) | 1.00  (ref) |  | 1.00  (ref) |  |  |  |  |
| Present | | 132 (44.9%) | 201 (46.3%) | 333 (45.7%) | 1.01 (0.74-1.38) |  | 0.68 (0.47-0.97) |  |  |  |  |
| **Usual ductal hyperplasia** | |  |  |  |  | 0.129 |  | 0.592 |  |  |  |
| None | | 201 (68.4%) | 274 (63.1%) | 475 (65.2%) | 1.00  (ref) |  | 1.00  (ref) |  |  |  |  |
| Mild | | 24 (8.2%) | 39 (9.0%) | 63 (8.7%) | 1.50 (0.84-2.68) |  | 0.83 (0.29-2.35) |  |  |  |  |
| Moderate | | 60 (20.4%) | 96 (22.1%) | 156 (21.4%) | 1.36 (0.92-2.03) |  | 0.63 (0.25-1.51) |  |  |  |  |
| Florid | | 9 (3.1%) | 25 (5.8%) | 34 (4.7%) | 2.05 (0.89-4.73) |  | 0.62 (0.26-1.51) |  |  |  |  |
| **Calcifications** | |  |  |  |  | 0.603 |  | 0.661 |  |  |  |
| Absent | | 131 (44.6%) | 196 (45.2%) | 327 (44.9%) | 1.00  (ref) |  | 1.00  (ref) |  |  |  |  |
| Present | | 163 (55.4%) | 238 (54.8%) | 401 (55.1%) | 1.09 (0.79-1.51) |  | 0.92 (0.64-1.33) |  |  |  |  |
| **Fibroadenoma** | |  |  |  |  | 0.488 |  |  |  |  |  |
| Absent | | 199 (67.7%) | 310 (71.4%) | 509 (69.9%) | 1.00  (ref) |  |  |  |  |  |  |
| Present | | 95 (32.3%) | 124 (28.6%) | 219 (30.1%) | 0.89 (0.63-1.25) |  |  |  |  |  |  |
| **Intra-ductal papilloma** | |  |  |  |  | 0.304 |  |  |  |  |  |
| Absent | | 273 (92.9%) | 391 (90.3%) | 664 (91.3%) | 1.00  (ref) |  |  |  |  |  |  |
| Present | | 21 (7.1%) | 42 (9.7%) | 63 (8.7%) | 1.36 (0.76-2.42) |  |  |  |  |  |  |
| **Radial scars** | |  |  |  |  | 0.126 |  |  |  |  |  |
| Absent | | 283 (96.3%) | 402 (92.6%) | 685 (94.1%) | 1.00  (ref) |  |  |  |  |  |  |
| Present | | 11 (3.7%) | 32 (7.4%) | 43 (5.9%) | 1.77 (0.85-3.69) |  |  |  |  |  |  |
| **Duct Ectasia** | |  |  |  |  | 0.813 |  |  |  |  |  |
| Absent | | 273 (92.9%) | 401 (92.4%) | 674 (92.6%) | 1.00  (ref) |  |  |  |  |  |  |
| Present | | 21 (7.1%) | 33 (7.6%) | 54 (7.4%) | 1.08 (0.59-1.98) |  |  |  |  |  |  |
| **Mucocele like tumors** | |  |  |  |  | 0.106 |  |  |  |  |  |
| Absent | | 291 (99.0%) | 433 (99.8%) | 724 (99.5%) | 1.00  (ref) |  |  |  |  |  |  |
| Present | | 3 (1.0%) | 1 (0.2%) | 4 (0.5%) | 0.15 (0.02-1.49) |  |  |  |  |  |  |
| ^*^Adjusted for all covariates significant in age- and BMI- adjusted model from analysis of all women | | | | | | | | |  |  |  |
