# Supplementary material for: Association between mammographic breast density and histologic features of benign breast disease
Source: Breast Cancer Res. 2017 Dec 19;19:134. doi: 10.1186/s13058-017-0922-6 (PMC5735506; doi:10.1186/s13058-017-0922-6)
Supplement: Supplementary file 3 — Association between mammographic breast density and benign breast findings for the 2672 women with parenchymal pattern density measures. (DOCX 20 kb) [file 13058_2017_922_MOESM3_ESM.docx]

**Table S3.** Association between mammographic breast density and benign breast findings for the 2672 women with parenchymal pattern density measures.

|  | |  |  |  | **Age- and BMI-Adjusted** | | **Fully Adjusted*** | |  |  |  |  |
| --- | --- | --- | --- | --- | --- | --- | --- | --- | --- | --- | --- | --- |
| Covariate | | Non-Dense N=943 | Dense N=1729 | Total N=2672 | Odds Ratio  (95% CI) | p-value | Odds Ratio  (95% CI) | p-value |  |  |  |  |
| **Age of BBD** | |  |  |  |  | <0.001 |  | <0.001 |  |  |  |  |
| Mean (SD) | | 57.5 (12.9) | 51.3 (12.6) | 53.5 (13.0) | 0.96 (0.96-0.97) |  | 0.96 (0.95-0.97) |  |  |  |  |  |
| **BMI at biopsy** | |  |  |  |  | <0.001 |  | <0.001 |  |  |  |  |
| Mean (SD) | | 28.2 (5.9) | 25.6 (5.1) | 26.5 (5.5) | 0.92 (0.91-0.93) |  | 0.92 (0.91-0.94) |  |  |  |  |  |
| **HRT ever/never** | |  |  |  |  | 0.004 |  | 0.010 |  |  |  |  |
| No | | 416 (44.1%) | 657 (38.0%) | 1073 (40.2%) | 1.00  (ref) |  | 1.00  (ref) |  |  |  |  |  |
| Yes | | 527 (55.9%) | 1072 (62.0%) | 1599 (59.8%) | 1.28 (1.08-1.52) |  | 1.26 (1.06-1.50) |  |  |  |  |  |
| **ADH** | |  |  |  |  | 0.92 |  |  |  |  |  |  |
| Absent | | 920 (97.6%) | 1694 (98.0%) | 2614 (97.8%) | 1.00  (ref) |  |  |  |  |  |  |  |
| Present | | 23 (2.4%) | 35 (2.0%) | 58 (2.2%) | 1.03 (0.59-1.80) |  |  |  |  |  |  |  |
| **ALH** | |  |  |  |  | 0.007 |  | 0.108 |  |  |  |  |
| Absent | | 913 (96.8%) | 1648 (95.3%) | 2561 (95.8%) | 1.00  (ref) |  | 1.00  (ref) |  |  |  |  |  |
| Present | | 30 (3.2%) | 81 (4.7%) | 111 (4.2%) | 1.84 (1.18-2.87) |  | 1.47 (0.92-2.34) |  |  |  |  |  |
| **Involution** | |  |  |  |  | 0.032 |  | 0.008 |  |  |  |  |
| Complete | | 317 (33.6%) | 359 (20.8%) | 676 (25.3%) | 1.00  (ref) |  | 1.00  (ref) |  |  |  |  |  |
| Partial | | 537 (56.9%) | 1077 (62.3%) | 1614 (60.4%) | 1.28 (1.05-1.57) |  | 0.94 (0.75-1.17) |  |  |  |  |  |
| None | | 89 (9.4%) | 293 (16.9%) | 382 (14.3%) | 1.42 (1.03-1.97) |  | 1.50 (1.06-2.12) |  |  |  |  |  |
| **Fibrosis** | |  |  |  |  | <0.001 |  | <0.001 |  |  |  |  |
| Absent | | 384 (40.7%) | 385 (22.3%) | 769 (28.8%) | 1.00  (ref) |  | 1.00  (ref) |  |  |  |  |  |
| Present | | 559 (59.3%) | 1344 (77.7%) | 1903 (71.2%) | 2.52 (2.10-3.03) |  | 2.40 (1.96-2.93) |  |  |  |  |  |
| **CCH / FEA** | |  |  |  |  | <0.001 |  | 0.281 |  |  |  |  |
| Absent | | 683 (72.4%) | 1116 (64.5%) | 1799 (67.3%) | 1.00  (ref) |  | 1.00  (ref) |  |  |  |  |  |
| Present | | 260 (27.6%) | 613 (35.5%) | 873 (32.7%) | 1.71 (1.43-2.06) |  | 1.18 (0.88-1.58) |  |  |  |  |  |
| **Sclerosing Adenosis** | |  |  |  |  | <0.001 |  | 0.289 |  |  |  |  |
| absent | | 661 (70.1%) | 1049 (60.7%) | 1710 (64.0%) | 1.00  (ref) |  | 1.00  (ref) |  |  |  |  |  |
| present | | 282 (29.9%) | 680 (39.3%) | 962 (36.0%) | 1.63 (1.36-1.95) |  | 1.14 (0.89-1.46) |  |  |  |  |  |
| **Cyst** | |  |  |  |  | <0.001 |  | 0.138 |  |  |  |  |
| Absent | | 395 (41.9%) | 589 (34.1%) | 984 (36.8%) | 1.00  (ref) |  | 1.00  (ref) |  |  |  |  |  |
| Present | | 548 (58.1%) | 1140 (65.9%) | 1688 (63.2%) | 1.63 (1.37-1.94) |  | 1.17 (0.81-1.68) |  |  |  |  |  |
| **Usual ductal hyperplasia** | |  |  |  |  | <0.001 |  | 0.261 |  |  |  |  |
| None | | 549 (58.2%) | 960 (55.5%) | 1509 (56.5%) | 1.00  (ref) |  | 1.00  (ref) |  |  |  |  |  |
| Mild | | 155 (16.4%) | 275 (15.9%) | 430 (16.1%) | 1.11 (0.88-1.40) |  | 0.89 (0.60-1.32) |  |  |  |  |  |
| Moderate | | 167 (17.7%) | 355 (20.5%) | 522 (19.5%) | 1.60 (1.28-2.01) |  | 1.16 (0.81-1.68) |  |  |  |  |  |
| Florid | | 72 (7.6%) | 139 (8.0%) | 211 (7.9%) | 1.57 (1.13-2.17) |  | 1.11 (0.76-1.63) |  |  |  |  |  |
| **Calcifications** | |  |  |  |  | <0.001 |  | 0.243 |  |  |  |  |
| Absent | | 516 (54.7%) | 895 (51.8%) | 1411 (52.8%) | 1.00  (ref) |  | 1.00  (ref) |  |  |  |  |  |
| Present | | 427 (45.3%) | 834 (48.2%) | 1261 (47.2%) | 1.43 (1.20-1.70) |  | 1.12 (0.93-1.36) |  |  |  |  |  |
| **Fibroadenoma** | |  |  |  |  | 0.086 |  |  |  |  |  |  |
| Absent | | 665 (70.6%) | 1259 (72.8%) | 1924 (72.0%) | 1.00  (ref) |  |  |  |  |  |  |  |
| Present | | 277 (29.4%) | 470 (27.2%) | 747 (28.0%) | 0.85 (0.70-1.02) |  |  |  |  |  |  |  |
| **Intra-ductal papilloma** | |  |  |  |  | 0.207 |  |  |  |  |  |  |
| Absent | | 865 (92.2%) | 1599 (92.7%) | 2464 (92.5%) | 1.00  (ref) |  |  |  |  |  |  |  |
| Present | | 73 (7.8%) | 126 (7.3%) | 199 (7.5%) | 1.23 (0.89-1.69) |  |  |  |  |  |  |  |
| **Radial scars** | |  |  |  |  | 0.158 |  |  |  |  |  |  |
| Absent | | 883 (93.6%) | 1600 (92.6%) | 2483 (93.0%) | 1.00  (ref) |  |  |  |  |  |  |  |
| Present | | 60 (6.4%) | 127 (7.4%) | 187 (7.0%) | 1.27 (0.91-1.77) |  |  |  |  |  |  |  |
| **Duct Ectasia** | |  |  |  |  | 0.480 |  |  |  |  |  |  |
| Absent | | 792 (84.0%) | 1451 (84.0%) | 2243 (84.0%) | 1.00  (ref) |  |  |  |  |  |  |  |
| Present | | 151 (16.0%) | 276 (16.0%) | 427 (16.0%) | 1.09 (0.87-1.36) |  |  |  |  |  |  |  |
| **Mucocele like tumors** | |  |  |  |  | 0.218 |  |  |  |  |  |  |
| Absent | | 933 (98.9%) | 1699 (98.5%) | 2632 (98.7%) | 1.00  (ref) |  |  |  |  |  |  |  |
| Present | | 10 (1.1%) | 25 (1.5%) | 35 (1.3%) | 1.63 (0.75-3.56) |  |  |  |  |  |  |  |
| ^*^Adjusted for all covariates significant in age- and BMI- adjusted model from analysis of all women | | | | | | | | | |  |  |  |
